# Frequency and Demographic Patterns of SARS‐CoV‐2 and Influenza Virus Infections in Khuzestan: A Retrospective Observational Study (2021–2023)

**DOI:** 10.1155/av/9988311

**Published:** 2026-05-19

**Authors:** Sepideh Nasimzadeh, Reza Mohammadpour Fard, Somayeh Ghasemi, Farnaz Marhemati, Meysam Movahedi, Babak Rashtizadeh, Faleh Darvishi, Saeed Hesam, Manoochehr Makvandi, Ali Khodadadi, Mehdi Torabizadeh, Najmaldin Saki, Negin Balar, Mohammad Rashno

**Affiliations:** ^1^ Department of Virology, School of Medicine, Ahvaz Jundishapur University of Medical Sciences, Ahvaz, Iran, ajums.ac.ir; ^2^ Student Research Committee, Ahvaz Jundishapur University of Medical Sciences, Ahvaz, Iran, ajums.ac.ir; ^3^ Cellular and Molecular Research Center, Medical Basic Sciences Research Institute, Ahvaz Jundishapur University of Medical Sciences, Ahvaz, Iran, ajums.ac.ir; ^4^ Faculty of Pharmaceutical, Biomedical, and Veterinary Sciences, University of Antwerp, Antwerp, Belgium, uantwerpen.be; ^5^ Reference Laboratory, Deputy of Treatment, Ahvaz Jundishapur University of Medical Sciences, Ahvaz, Iran, ajums.ac.ir; ^6^ Imam Khomeini Hospital Clinical Research Development Unit, Ahvaz Jundishapur University of Medical Sciences, Ahvaz, Iran, ajums.ac.ir; ^7^ Department of Biostatistics and Epidemiology, School of Health, Ahvaz Jundishapur University of Medical Sciences, Ahvaz, Iran, ajums.ac.ir; ^8^ Department of Immunology, Ahvaz Jundishapur University of Medical Sciences, Ahvaz, Iran, ajums.ac.ir; ^9^ Golestan Hospital Clinical Research Development Unit, Ahvaz Jundishapur University of Medical Sciences, Ahvaz, Iran, ajums.ac.ir; ^10^ Thalassemia & Hemoglobinopathy Research Center, Health Research Institute, Ahvaz Jundishapur University of Medical Sciences, Ahvaz, Iran, ajums.ac.ir

**Keywords:** coinfection, COVID-19, flu, frequency, influenza virus, SARS-CoV-2 virus

## Abstract

**Objective:**

To describe the frequency and demographic characteristics of SARS‐CoV‐2 and influenza infections, as well as their co‐occurrence, among patients in Khuzestan province.

**Methods:**

This retrospective laboratory‐based study was conducted at the Reference Laboratory of Ahvaz Jundishapur University of Medical Sciences. Between March 2021 and November 2023, respiratory swab samples from patients with suspected Coronavirus disease 2019 (COVID‐19) or influenza referred from 64 affiliated healthcare institutions (23 health centers and 41 hospitals) were tested for SARS‐CoV‐2 and influenza A/B using RT‐qPCR. Demographic and laboratory data were analyzed using chi‐square and simple logistic regression, and the results were expressed as odds ratios (ORs) with 95% confidence intervals (CIs).

**Results:**

A total of 148,079 respiratory specimens were analyzed between 2021 and 2023. Among them, 41.2% tested positive for SARS‐CoV‐2 in 2021, 14.9% in 2022, and 4.5% in 2023, showing a marked decline in COVID‐19 positivity over time. In contrast, influenza positivity progressively increased from 13.3% in 2021 to 15.5% in 2022 and 20.8% in 2023, coinciding with broader testing coverage. Females consistently showed higher infection rates for both viruses. The most affected age groups for COVID‐19 shifted from 40 to 50 years in 2021 to 20–30 years in 2022 and ≥ 70 years in 2023, while influenza was more frequent among individuals under 20 years in 2023. Coinfection with SARS‐CoV‐2 and influenza was uncommon, detected in only 0.6% of the patients in 2023, predominantly involving the H1N1 subtype.

**Conclusion:**

The present study demonstrates a declining trend of SARS‐CoV‐2 infection alongside a progressive rise in influenza cases in southwest Iran from 2021 to 2023. The findings highlight age‐ and sex‐related variations in infection patterns and underscore the value of continuous dual surveillance for both viruses.

## 1. Introduction

Coronavirus disease 2019 (COVID‐19), caused by the severe acute respiratory syndrome coronavirus 2 (SARS‐CoV‐2), is a highly contagious disease that originated in Wuhan, China, in December 2019 [1]. It has since become a global epidemic, with over 775 million reported cases and 7 million deaths worldwide [2]. In addition to the direct health burden, COVID‐19 has contributed to a rise in secondary complications such as anxiety, depression, diabetes, cardiovascular diseases, and chronic kidney disorders, significantly diminishing the quality of life among affected individuals [3–5].

Despite global vaccination efforts and improvements in disease surveillance, COVID‐19 continues to pose challenges due to emerging variants and recurrent outbreaks [6]. During the same period, influenza viruses have persisted as major causes of respiratory infections worldwide, accounting for approximately 1 billion infections and up to 650,000 deaths annually [7]. The concurrent circulation of SARS‐CoV‐2 and seasonal influenza viruses (types A and B) has complicated diagnostic and clinical management, as both share similar modes of transmission and present with overlapping clinical manifestations, such as fever, cough, shortness of breath, chills, sore throat, runny nose, fatigue, headache, and body pain [8]. The pooled global coinfection rate of influenza among patients with SARS‐CoV‐2 infection lies in the range of approximately 0.5%–20% though this rate varies by region and testing policy [9, 10]. Regional studies from Asia and the Middle East have also demonstrated that dual infection can exacerbate disease severity, prolong hospitalization, and increase the risk of complications such as acute respiratory distress syndrome (ARDS) and mortality [11, 12]. Therefore, routine molecular testing for both viruses in patients with suspected respiratory infection is crucial to reduce misdiagnosis, identify dual infection, and mitigate risk of severe complications.

In Iran, and particularly in the southwestern provinces, limited data exist on the epidemiology of both viruses in the postvaccination era. Characterizing the temporal and demographic patterns of these infections across consecutive years is essential for understanding postpandemic shifts in respiratory disease dynamics and guiding vaccine policy optimization. Accordingly, this study aimed to describe the frequency and demographic distribution of SARS‐CoV‐2 and influenza virus infections, as well as their co‐occurrence, in Khuzestan province between 2021 and 2023.

## 2. Material and Methods

### 2.1. Study Design and Ethical Approval

This retrospective, laboratory‐based observational study was conducted at the Reference Laboratory of Ahvaz Jundishapur University of Medical Sciences (AJUMS), located in Ahvaz, Khuzestan province, southwest Iran. The laboratory serves as a tertiary‐level diagnostic center and the regional referral site for molecular detection of respiratory viruses, including SARS‐CoV‐2 and influenza. The study period covered March 2021–November 2023. Ethical approval for this study was obtained from the Research Ethics Committee (IR.AJUMS.REC.1402.051), and institutional authorization was granted for the use of anonymized laboratory records.

### 2.2. Study Population and Data Source

All patients presenting with respiratory symptoms who were clinically suspected of having COVID‐19 or influenza infection during the study period were included. Common symptoms of viral respiratory infections, such as fever above 38°C, sore throat, cough, dyspnea, malaise, and other typical influenza‐like manifestations were considered as inclusion criteria. Nasopharyngeal and oropharyngeal swab samples were obtained from both inpatients and outpatients referred by 64 affiliated healthcare facilities, including 23 health centers and 41 hospitals across Khuzestan province.

The collected specimens were transported to the Reference Laboratory in Viral Transport Medium (VTM) under cold‐chain conditions maintained at 4°C and processed immediately upon receipt for molecular testing. Detection of SARS‐CoV‐2 and influenza viruses was performed using reverse transcription–quantitative polymerase chain reaction (RT‐qPCR) assays in accordance with the laboratory’s standardized diagnostic protocols. Data were retrieved from the laboratory’s electronic medical record system and included patient age, sex, year of sampling, infection type (SARS‐CoV‐2, influenza A/B, or coinfection), and PCR result. Exclusion criteria comprised duplicate entries and inconclusive PCR results.

### 2.3. RNA Extraction and RT‐qPCR

RNA extraction and RT‐qPCR are routinely performed at the Reference Laboratory. Viral RNA was extracted using either the BehPreb Viral Nucleic Acid Extraction Kit (Behgen, Iran) or the Zybio Viral Nucleic Acid Extraction Kit (Zybio, China). Automated extraction was carried out using the Automatic Nucleic Acid Isolation System EXM3000 (Zybio, China) according to the manufacturer’s instructions. For each extraction, confirmed positive and negative samples served as the control samples.

For SARS‐CoV‐2 detection, amplification was performed using the COVID‐19 One‐Step RT‐PCR Kit and SARS‐FLU Plus One‐Step RT‐PCR Kit (Pishtaz Teb, Iran) or the Covitech COVID‐19 One‐Step RT‐PCR Kit (Atiye Bahman, Iran), which include viral gene‐specific primers. For influenza A and B, testing followed the National Influenza Center of Iran protocols. When the diagnostic kit did not include influenza primers, the Center’s approved primer sets were employed for influenza typing and subtyping. Each reaction contained 10 µL of extracted RNA mixed with 10 µL of reaction buffer containing primers and probes specific for influenza A/B detection.

Amplification and fluorescence detection were performed using either the Roche LightCycler 96 Instrument (Roche, Germany) or the QIAquant Real‐Time PCR System (Qiagen, Germany). Both instruments undergo annual calibration using manufacturer‐provided calibration kits. The calibration process includes running control samples and standards to ensure that the instruments meet required performance criteria, including optimal reaction efficiency and cycle threshold (CT) value consistency across runs. To ensure quality control, each RT‐qPCR run included positive controls, negative controls, and nontemplate controls (NTCs) to verify assay validity. Additionally, the RNase P gene was utilized as an internal control during both RNA extraction and PCR amplification. This gene provided valuable feedback on sample collection, extraction efficiency, and the quality of PCR amplification. The PCR results were interpreted by the Laboratory Supervisor, and all analyses were performed by the Statistician (S.H.).

### 2.4. Statistical Analysis

Statistical analyses were performed using SPSS software, Version 24 (IBM Corp., Armonk, NY, USA). Descriptive statistics (frequency and percentage) were used to summarize demographic and laboratory characteristics, including age group, sex, infection type (SARS‐CoV‐2, influenza A/B, or coinfection), and year of testing (2021–2023). Associations between categorical variables were examined using the chi‐square test. Additionally, the odds ratio (OR) with 95% confidence interval (CI) was calculated to quantify the strength of associations. A *p* value < 0.05 was considered statistically significant.

To assess the relationship of the demographic factors with infection status, simple (univariable) binary logistic regression models were fitted separately for each independent variable. Specifically, for each outcome (SARS‐CoV‐2 positive vs. negative; influenza positive vs. negative), we fitted individual logistic regression models with either age group (categorized as < 20, 20–30, 30–40, 40–50, 50–60, 60–70, > 70 years; reference category: < 20 years) or sex (male/female) as the independent variable.

## 3. Results

A total of 148,079 subjects were assessed for influenza or COVID‐19 infection between 2021 and 2023. Of these, 94,589 (63.9%) samples were collected from March 2021 to March 2022, 30,050 (20.3%) from March 2022 to March 2023, and 23,440 (15.8%) from March 2023 to November 2023.

### 3.1. Frequency of COVID‐19

During the first study period (March 2021–March 2022), 91,544 patients were evaluated for COVID‐19 infection and 42.5% of the tested individuals were positive for SARS‐CoV‐2. The infection rate was higher among females (48.0%) than males (38.9%), and the highest positivity was observed in the 40–50‐year age group, whereas the < 20‐year group had the lowest detection rate.

**FIGURE 1:**
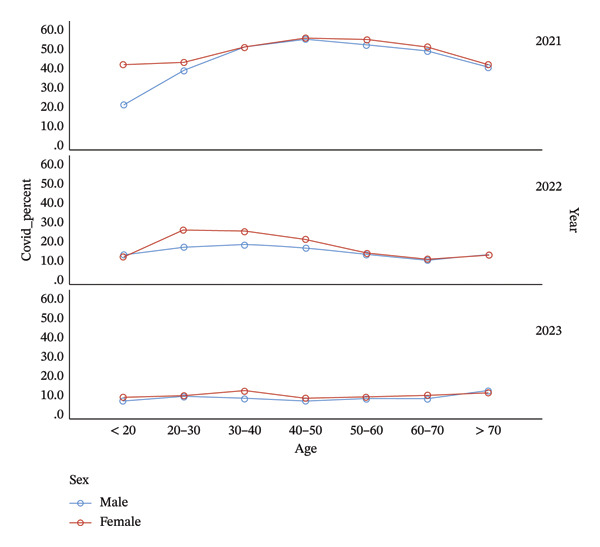
The frequency and demographic patterns of COVID‐19 infection between 2021 and 2023 in Khuzestan.

In the second period (March 2022–March 2023), the overall positivity for SARS‐CoV‐2 declined to 14.9%, again showing a higher frequency in females (16.9%) than in males (13.3%). The most affected age groups were 20–30 years and 30–40 years, both exceeding 21% positivity (Table 1 and Figure 1).

**TABLE 1:** The frequency of COVID‐19 between March 2021 and March 2023.

Variables	Subgroups	March 2021–March 2022		March 2022–March 2023	
		Negative (%)	Positive (%)	Negative (%)	Positive (%)
Sex	Male	33,440 (61.1)	21,323 (38.9)	12,046 (86.7)	1853 (13.3)
	Female	19,112 (52)	17,669 (48)	8834 (83.1)	1795 (16.9)

Age	< 20	17,588 (74.1)	6142 (25.9)	4349 (87.7)	608 (12.3)
	20–30	5281 (59.5)	3592 (40.5)	1838 (78.5)	502 (21.5)
	30–40	7305 (49.8)	7370 (50.2)	2658 (78.6)	722 (21.4)
	40–50	5455 (45.1)	6631 (54.9)	2249 (82)	493 (18)
	50–60	4928 (47.5)	5454 (52.5)	2453 (87.1)	364 (12.9)
	60–70	5132 (50.5)	5035 (49.5)	2989 (89.9)	334 (10.1)
	> 70	6863 (59)	4768 (41)	4344 (78.4)	625 (12.6)

Total		52,552 (57.5)	38,992 (42.5) ‬	20,880 (‬85.1)	3648 (14.9)

Between March 2023 and November 2023, a total of 12,122 individuals were tested, of whom 8.7% were positive for SARS‐CoV‐2. The infection rate remained significantly higher in females than in males (*p* = 0.037). Additionally, subjects aged 30–40 years (9.5%) and those over 70 years (10.7%) had significantly higher infection rates (Table 2 and Figure 1).

**TABLE 2:** The frequency of COVID‐19 between March 2023 and November 2023.

Variables	Subgroups	Negative (%)	Positive (%)	B	OR	95% CI	*p* value
Sex	Male	6582 (91.7) ‬	597 (8.3)	ref	ref	ref	ref
	Female	4478 (90.6) 4943‬	465 (9.4)	0.14	1.15	(1.09,1.30)	0.037

Age	< 20	2372 (92.8)	185 (7.2)	ref	ref	ref	ref
	20–30	678 (90.8)	69 (9.2)	0.27	1.311	(0.98,1.74)	0.071
	30–40	1197 (90.5)	125 (9.5)	0.29	1.34	(1.06,1.7)	0.016
	40–50	1233 (92.4)	101 (7.6)	0.05	1.05	(0.82,1.35)	0.70
	50–60	1309 (91.8)	117 (8.2)	0.14	1.15	(0.9,1.46)	0.268
	60–70	1728 (91.6)	159 (8.4)	0.16	1.18	(0.95,1.47)	0.142
	> 70	2543 (89.3)	306 (10.7)	0.43	1.54	(1.28,1.87)	< 0.001

### 3.2. Frequency of Influenza

During the first study period (March 2021–March 2022), 3045 patients were examined for influenza infection and 13.3% of the tested patients were positive. The occurrence of this infection was consistent between males and females (13.6% and 13%, respectively) and the highest positivity was observed in the 20–29‐year age group.

**FIGURE 2:**
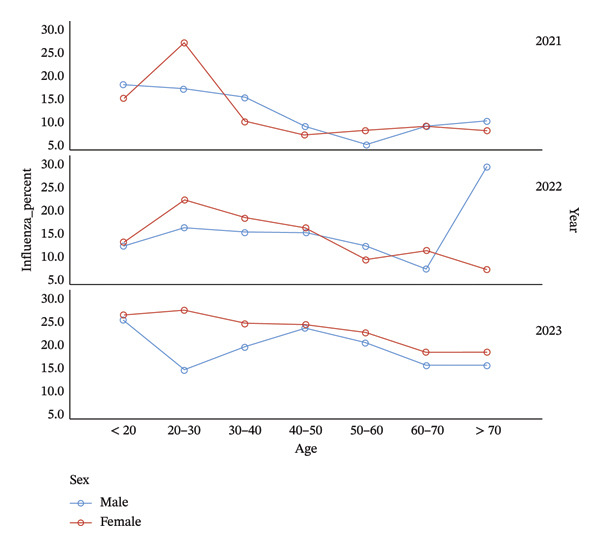
The frequency and demographic patterns of influenza infection between 2021 and 2023 in Khuzestan.

In the following year (March 2022–March 2023), the influenza positivity rate increased to 15.5%. Interestingly, males (17.3%) were found to be more affected by influenza than females (12.9%), and older adults (≥ 70 years) and young adults (20–29 years) exhibited the greatest infection rates (21.9% and 19.7%, respectively) (Table 3 and Figure 2).

**TABLE 3:** The frequency of influenza between March 2021 and March 2023.

Variables	Subgroups	March 2021–March 2022		March 2022–March 2023	
		Negative (%)	Positive (%)	Negative (%)	Positive (%)
Sex	Male	1515 (86.4)	238 (13.6)	2761 (82.7)	576 (17.3)
	Female	1124 (87)	168 (13)	1904 (87.1)	281 (12.9)

Age	< 20	813 (82.4)	174 (17.6)	1146 (87.4)	165 (12.6)
	20–30	200 (76.9)	60 (23.1)	265 (80.3)	65 (19.7)
	30–40	268 (87.3)	39 (12.7)	460 (83.2)	93 (16.8)
	40–50	252 (91.6)	23 (8.4)	420 (84.2)	79 (15.8)
	50–60	261 (93.5)	18 (6.5)	532 (89)	66 (11)
	60–70	301 (90.4)	32 (9.6)	711 (90.8)	72 (9.2)
	> 70	544 (90.1)	60 (9.9)	1131 (78.1)	317 (21.9)

Total		2639‬ (86.7)	406 (13.3)	4665‬ (84.5)	857‬ (15.5)

Between March and November 2023, 11,318 patients were tested, of whom 20.8% were positive. The infection rate was significantly higher among females than males (*p* < 0.001). The < 20‐year age group showed the highest positivity, while the 60–70‐year group had the lowest detection rate (Table 4 and Figure 2).

**TABLE 4:** The frequency of influenza between March 2023 and November 2023.

Variables	Subgroups	Negative (%)	Positive (%)	B	OR	95% CI	*p* value
Sex	Male	5400 (80.7)	1293 (19.3)	Ref	Ref	Ref	Ref
	Female	3565 (77.1)	1060 (22.9)	0.21	1.24	(1.13,1.36)	< 0.001

Age	< 20	1781 (74)	625 (26)	0.58	1.79	(1.57,2.05)	< 0.001
	20–30	562 (78.9)	150 (21.1)	0.31	1.36	(1.11,1.67)	0.003
	30–40	952 (78.2)	266 (21.8)	0.36	1.43	(1.21,1.69)	< 0.001
	40–50	957 (78.2)	299 (23.8)	0.47	1.6	(1.36,1.88)	< 0.001
	50–60	1143 (78.4)	315 (21.6)	0.34	1.41	(1.2,1.65)	< 0.001
	60–70	1169 (83.7)	228 (16.3)	−0.01	0.99	(0.84,1.18)	0.967
	> 70	2401 (83.6)	470 (16.4)	ref	ref	ref	ref

### 3.3. Coinfection Rate of COVID‐19 and Influenza

From March 2021 to March 2022, three individuals, comprising two men and one woman, were diagnosed with a coinfection of influenza and COVID‐19, with all patients infected with Flu type B. During the period from March 2022 to March 2023, eight subjects, consisting of two females (25%) and six males (75%), were identified as having a coinfection with both Flu type B and Flu type A (H3 subtype).

Further analysis disclosed that 0.6% (66) of the tested patients were positive for coinfection of COVID‐19 and influenza between March and November 2023 (*p* value < 0.001) (Table 5). Out of these patients, 60.6% were females and 39.4% were males. In 2023, 72.7% of the patients were infected with the H1N1 subtype, while 27.3% were infected with the H3N2 subtype. Furthermore, 18.2% of the participants had an unknown influenza type, and only one patient (1.5%) had Type B.

**TABLE 5:** Coinfection rate of COVID‐19 and influenza between March and November 2023.

F	C		
	Negative (%)	Positive (%)	*p* value
Negative	7969 (70.4)	996 (8.8)	< 0.001
Positive	2287 (20.2)	66 (0.6)	

*Note:* C: COVID‐19, F: flu.

## 4. Discussion

### 4.1. Interpretation of Findings

In this study, we analyzed the frequency and demographic distribution of SARS‐CoV‐2 and influenza infections based on 148,079 specimens across Khuzestan province between 2021 and 2023. The results revealed a clear inverse trend between the two viruses: while the positivity rate of SARS‐CoV‐2 declined from 41.2% in 2021 to 4.5% in 2023, influenza detection increased from 13.3% to 20.8% during the same period. These findings align with postpandemic global observations indicating a resurgence of seasonal influenza activity following the relaxation of COVID‐19‐related public‐health restrictions [13]. Several contextual factors may explain this divergence. During the pandemic, the national healthcare system prioritized COVID‐19 prevention and control through extensive testing and free mass vaccination campaigns. In contrast, influenza vaccination remained optional and self‐funded, resulting in lower immunization rates across the population. Moreover, in the early phase of the pandemic, laboratory resources were primarily allocated to SARS‐CoV‐2 detection, limiting routine influenza testing. With the introduction of multiplex molecular diagnostic panels capable of simultaneously identifying SARS‐CoV‐2, influenza, and other respiratory pathogens, influenza testing became more accessible and comprehensive. Consequently, the progressive increase in influenza detection after 2021 likely reflects both enhanced diagnostic capacity and the gradual restoration of its circulation as COVID‐19 containment measures were lifted.

The age‐specific distribution of infections in this study revealed dynamic patterns over the three‐year period. COVID‐19 was most prevalent among individuals aged 40–50 years in 2021 (*p* < 0.001), but the burden progressively shifted toward younger adults (20–40 years) in 2022 and older adults (≥ 70 years) in 2023. This transition may be associated with vaccination prioritization of older adults during the initial immunization campaigns, followed by waning immunity in later years. Conversely, influenza infection was most frequent among younger populations (< 20 years) in 2023 (*p* < 0.001), consistent with reports from Asia showing that school‐aged individuals play a key role in influenza transmission [14]. Additionally, the consistently higher positivity rate among females for both SARS‐CoV‐2 and influenza observed in our data aligns with previous Iranian and regional studies [15, 16]. This pattern may be related to differences in healthcare‐seeking behavior or biological susceptibility influenced by hormonal and immunological factors.

Our data showed that coinfections with SARS‐CoV2 and flu viruses were low among all patients. This finding is consistent with Ozaras et al.’s research, where only 6 out of 1103 COVID‐19 patients were coinfected with influenza [17]. Both COVID‐19 and influenza infections can occur in the same patient and present with similar symptoms, such as fever, cough, and shortness of breath. However, common symptoms for influenza infection include fatigue, headache, and myalgia, while COVID‐19 primarily presents with cough and shortness of breath accompanied by fever. Another investigation in Wuhan showed that only 5 out of 115 COVID‐19 patients were coinfected with flu, and pharyngeal pain was more prevalent in these coinfected patients [18]. To better understand the impact of influenza infection on COVID‐19 progression, Hu et al. compared anti‐influenza A virus IgM‐positive and ‐negative COVID‐19 patients. They found that coinfection with influenza A virus was significantly more frequent in female COVID‐19 patients [19]. Regarding subtypes, our study demonstrated that all influenza A–positive patients were subtyped as either H1 (72.7%) or H3 (27.3%). This subtype distribution closely parallels the findings of Hashemi et al. in northeastern Iran, where H1N1 accounted for 78.3% of the cases [20]. The predominance of H1 in both studies may reflect its continued circulation as the dominant seasonal strain in Iran during the postpandemic years, possibly facilitated by partial cross‐protection from prior exposure and suboptimal influenza vaccination coverage in the general population.

### 4.2. Limitation

This study has several limitations that should be considered. First, the study did not examine all patients for both influenza and SARS‐CoV‐2. This is because there were no simultaneous diagnosis kits for COVID‐19 and influenza, and influenza diagnosis tests were only conducted on samples requested by specialists for hospitalized patients in 2021 and 2022. Due to the large number of samples from health centers, influenza diagnostic tests were limited to samples from hospitalized patients, in addition to COVID‐19 diagnostic tests. Consequently, influenza data from those years may underestimate the true infection frequency in the general population, whereas the 2023 dataset provides a more reliable estimate of concurrent viral circulation. Second, information such as previous infection history, comorbidities, and vaccination status (for both influenza and COVID‐19), which would have provided more reliable results, was not available. Future studies incorporating clinical and immunological data are warranted to better characterize the dynamics of respiratory virus co‐circulation in this population.

## 5. Conclusion

This retrospective analysis provides an overview of SARS‐CoV‐2 and influenza virus activity across Khuzestan province from 2021 to 2023, revealing a distinct inverse relationship between their circulation patterns. The marked decline in SARS‐CoV‐2 detection and the parallel rise in influenza incidence reflect postpandemic shifts in population immunity, testing practices, and viral competition within the respiratory ecosystem. The observed demographic variations—particularly the higher infection rates among females and the changing age distribution across study years—underscore the influence of behavioral, biological, and vaccination‐related factors on infection dynamics. Although the rate of SARS‐CoV‐2 and influenza coinfection was low, continuous molecular monitoring remains essential for early detection and effective outbreak management. Strengthening dual surveillance and improving vaccination coverage for both viruses could help optimize regional preparedness against future respiratory epidemics.

## Author Contributions

Mohammad Rashno, Somayeh Ghasemi, Farnaz Marhemati, Meysam Movahedi, Babak Rashtizadeh, and Faleh Darvishi collaborated in performing molecular testing of the patients’ samples and data collection. Mohammad Rashno, Saeed Hesam, Sepideh Nasimzadeh, Reza Mohammadpour Fard, Negin Balar, and Somayeh Ghasemi analyzed the patient’s data or wrote the manuscript. Manoochehr Makvandi, Ali Khodadadi, Mehdi Torabizadeh, and Najmaldin Saki reviewed the manuscript and provided consultation regarding intellectual argumentation.

## Funding

This study was approved by Research Affairs, Ahvaz Jundishapur University of Medical Sciences, Ahvaz, Iran (no: CMRC‐0204). The authors received no financial support for the research, authorship, and publication of this article.

## Ethics Statement

Ethical approval was obtained from the Research Ethics Committee at University of Ahvaz Jundishapur University of Medical Sciences, Ahvaz, Iran (IR.AJUMS.REC.1402.051).

## Conflicts of Interest

The authors declare no conflicts of interest.

## Data Availability

All data were collected from the reference laboratory’s electronic medical record system and the data that support the findings of this study are available from Reference Laboratory Ahvaz Jundishapur University of Medical Sciences, Ahvaz, Iran, but restrictions apply to the availability of these data, which were used under license for this study, and so are not publicly available. Data are available with the permission of reference laboratory.
